# Genetic Variation in Root Architectural Traits in *Lactuca* and Their Roles in Increasing Phosphorus-Use-Efficiency in Response to Low Phosphorus Availability

**DOI:** 10.3389/fpls.2021.658321

**Published:** 2021-05-03

**Authors:** Amira Beroueg, François Lecompte, Alain Mollier, Loïc Pagès

**Affiliations:** ^1^PSH Unit, INRAE, F-84914, Avignon, France; ^2^ISPA Unit, Bordeaux Sciences Agro, INRAE, Villenave d’Ornon, France

**Keywords:** *Lactuca*, phosphorus, Root architectural traits, phosphorus-use-efficiency, maximum apical diameter, root biomass, genotypes variations

## Abstract

Low phosphorus (P) bioavailability in the soil and concerns over global P reserves have emphasized the need to cultivate plants that acquire and use P efficiently. Root architecture adaptation to low P can be variable depending on species or even genotypes. To assess the genetic variability of root architectural traits and their responses to low P in the *Lactuca* genus, we examined fourteen genotypes including wild species, ancient and commercial lettuce cultivars at low (LP, 0.1 mmol. L^–1^) and high P (HP, 1 mmol. L^–1^). Plants were grown in cylindrical pots adapted for the excavation and observation of root systems, with an inert substrate. We identified substantial genetic variation in all the investigated root traits, as well as an effect of P availability on these traits, except on the diameter of thinner roots. At low P, the main responses were a decrease in taproot diameter, an increase in taproot dominance over its laterals and an increase in the inter-branch distance. Although the genotype x P treatment effect was limited to root depth, we identified a tradeoff between the capacity to maintain a thick taproot at low P and the dominance of the taproot over its laterals. Regardless of the P level, the phosphorus-use-efficiency (PUE) varied among lettuce genotypes and was significantly correlated with total root biomass regardless of the P level. As taproot depth and maximum apical diameter were the principal determinants of total root biomass, the relative increase in PUE at low P was observed in genotypes that showed the thickest apical diameters and/or those whose maximal apical diameter was not severely decreased at low P availability. This pre-eminence of the taproot in the adaptation of *Lactuca* genotypes to low P contrasts with other species which rely more on lateral roots to adapt to P stress.

## Introduction

Phosphorus (P) is an important plant macronutrient and a component in various molecules such as ATP, nucleic acid, phospholipids and phosphoproteins. Phosphorus also plays an important role in an array of processes, including photosynthesis, energy generation, enzyme activation and inactivation (Vance et al., 2003). Plants absorb P through their roots mainly as orthophosphate ions H_2_PO_4_^–^ and HPO_4_^2–^, which can be tightly adsorbed into soil particles, form insoluble complexes with cations, and have poor mobility in the soil solution (Shen et al., 2011). As a consequence, P availability can be limited, which leads to the use of high amounts of mineral P fertilizers by farmers to overcome this limitation and sustain high crop yields. However, P fertilizer recovery is usually low, and an excess of P application following runoff or erosion can foster environmental damage such as water eutrophication (Shen et al., 2011; Sattari et al., 2016). Moreover, P fertilizers mostly derived from phosphate rocks and P is considered a non-renewable resource whose global reserve may be depleted in the future (Cordell et al., 2009). A more careful exploitation of P is required and one strategy is to increase P efficiency and recovery using plants, including varieties that are able to produce high yields with low P availability (Singh Gahoonia and Nielsen, 2004; Hammond et al., 2009; van de Wiel et al., 2016). Lettuce, *Lactuca sativa* L., is an important leafy vegetable, that is primarily consumed fresh in salads. As a cash crop that requires intensive labor at planting and harvest, lettuce is mostly cultivated in conventional systems that maximize yield and quality by supplying abundant water and nutrients, including P (Gallardo et al., 1996; Hoque et al., 2010; Kerbiriou et al., 2013). Phosphorus-use-efficiency (PUE) is generally defined as the amount of biomass or yield per unit of applied P (g dry mass/g applied P). PUE can be separated into the product of uptake efficiency (PUpE) (g P/g applied P) and utilization efficiency (PUtE) (g dry mass/g P). PUpE is based on the ratio pf P uptake in plants to the amount of P applied, and PUtE represents the production of plant biomass per unit P accumulated in the plant (Gourley et al., 1994).

The intensity of plant responses to low P availability varies among and within species (Abdolzadeh et al., 2010; Balemi, 2010; Rose and Wissuwa, 2012). Intraspecific variations of PUE and its components have been observed in many species such as rice (Vejchasarn et al., 2016), wheat (Bilal et al., 2018), barley (Wang et al., 2015), maize (Parentoni and De Souza Júnior, 2008), potato (Wacker-Fester et al., 2019) and the common bean (da Silva et al., 2014). A common pattern is an increase in PUE and its components (PUpE and PUtE) under P deficiency, which is made possible by several adaptive mechanisms. Plants can adapt to low P via several physiological mechanisms, and higher exudation of organic acids and enzymes, such as phosphatases and phytases, is commonly observed to facilitate the solubilization of P complexes and organic P in the rhizosphere (Balemi and Negisho, 2012; Minemba et al., 2019).

Plants can also improve P uptake through symbiotic associations with mycorrhizal fungi, whose hyphae increase the soil volume that can be explored (Campos et al., 2018). Plants may also improve P utilization by recycling internal P, remobilizing P from mature to young developing organs and reprioritizing metabolic P utilization in plants (Veneklaas et al., 2012; Irfan et al., 2020).

In response to low P, plants also adapt their function, such as carbon assimilation and allocation. A common feature is the increase in the root/shoot ratio under P deficiency and then an increased allocation of carbohydrates to the root system (Zhou et al., 2016; De Souza Campos et al., 2019). For example, tomato plants are able to recapture the carbon lost from root exudates, such as citrate and malate, to increase the translocation of carbon to shoots (Tiziani et al., 2020).

Plants with larger root systems have access to a greater soil volume and may recover more P (Lynch and Brown, 2008). Increased P acquisition can also be achieved by altering root morphology and architecture. The root system architecture, as the length, direction and spatial distribution of roots, is highly plastic and varies according to the genotype and environmental conditions (Lynch and Brown, 2008).

In many soils, P tends to be more available in the topsoil; therefore, in cases of P deficiency plants prefer a shallow root system that grows outward rather than downward. This “topsoil foraging” strategy is well observed in common bean (Ramaekers et al., 2010). The architectural traits associated with this topsoil exploration are the growth angle of basal roots, the dispersion of basal root lateral branching and the production of adventitious roots (Bonser et al., 1996; Lynch, 2007; Niu et al., 2013). Another widespread adaptation to low P is the modification of root hair length and density (Gahoonia and Nielsen, 1998; Brown et al., 2012).

In *Arabidopsis*, low P availability induced a highly branched root system with reduced primary root growth (López-Bucio et al., 2003). In lentils, P deficiency caused a significant increase in total root length and lateral root number (Sarker and Karmoker, 2009). However, such responses are not universal and the lateral root density has been shown to decrease under low P availability in several plant species such as rice (Vejchasarn et al., 2016) and the common bean (Borch et al., 1999). Such apparent discrepancies among root trait responses to low P could be explained by the phenotyping method, the intensity of P stress and other environmental conditions and/or the genetic material used.

Global, cumulative, or average root traits, such as total root length, root number, or average diameter, are not well suited to study the developmental path by which a plant adapts to nutrient deficiency. In addition, complex variables, such as specific root length, which aggregates information on the length, diameter, and density of a root, are also parameters not easy to interpret. Traits directly related to developmental processes should preferably be registered. If contrasting root architectural responses can be observed between different genera, less information is available regarding intraspecific or intragenus variability. In lettuce, although data on PUE are available (Buso and Bliss, 1988; Neocleous and Savvas, 2019), information on genetic variability is scarce, and to our knowledge, no information has been published regarding the plasticity of lettuce root system architecture in response to P nutrition and its impact on P acquisition.

This study aimed to characterize the intragenus variability of root system adaptation to variable P availability in *Lactuca*. We examined a panel of 14 *Lactuca* genotypes belonging to old and modern cultivars as well as wild-associated species. Traits reflecting the developmental process of the root system, including root elongation, root diameter and branching density, were observed. Six root traits were considered, and Pagès and Kervella (2018) indicated that they summarize the essential root architecture traits connected to the exploration and exploitation capacities of the root system.

The specific aims of this work were to (1) assess the genetic variability of root trait architecture under low- and high-P conditions, (2) investigate the range of plasticity of each trait and (3) evaluate their contribution to the PUE and its components.

## Materials and Methods

### Plant Material

Fourteen *Lactuca* genotypes were selected to represent diverse geographic origins and aerial phenotypes, which will likely present variability in the root system architecture. Wild species and ancient and modern genotypes of different lettuce types were selected (Table 1).

**TABLE 1:** Species, type, genotype name and origin of the lettuce used in this study.

No.	species	Type	Name of cultivar	Origin
1	*L. serriola*	Wild	LS367	Public domain
2	*L. saligna*	Wild	CR17	Public domain
3	*L. sativa*	Wild	PI251245	Public domain
4	*L. sativa*	Butterhead	Nenufar	Seed company (Vilmorin)
5	*L. sativa*	Butterhead	Bourguignonne	Public domain
6	*L. sativa*	Loose-leaf	Kilervi	Seed company (Rijk Zwaan)
7	*L. sativa*	Loose-leaf	Feuille de chêne blonde	Public domain
8	*L. sativa*	Romaine	Blonde lente à monter	Public domain
9	*L. sativa*	Butterhead	Green Mignonette	Public domain
10	*L. sativa*	Crisphead	De Pierre Bénite	Public domain
11	*L. sativa*	Crisphead	Ferega	Seed company (Enza Zaden)
12	*L. sativa*	Butterhead	Joviale	Seed company (Gautier)
13	*L. sativa*	Romaine	Socca	Seed company (Gautier)
14	*L. sativa*	Stem lettuce	Red Orient	Public domain

### Experimental Design

The experiment was carried out under a plastic tunnel at the national research institute for agriculture, food and the environment (INRAE) in Avignon, France, from October to November 2018. Seeds were sown in a rock wool cell placed in a growth chamber at 20/17°C day/night and 85% relative humidity. Ten days after seeding, uniform plantlets were transplanted in the tunnel into cylindrical PVC tubes (1 m in height and 10 cm diameter) in a grid that allowed drainage at the bottom. The substrate was a P-inert 1:1(V/V) mixture of vermiculite and river sand. A total of 84 tubes, each containing one plant, were set up, thus corresponding to three biological replications for each variety and two P treatments.

Water and fertilizers were supplied using a drip system controlled by a programmer. The substrate water content was controlled using soil moisture sensors (EC-5 Decagon, three per block placed in randomly selected tubes) and maintained close to field capacity throughout the experiment.

During the first week, all the plants were fertirrigated with a nutrient solution at a low P concentration (0.1 mmol. L^–1^ of P supplied with KH_2_PO_4_^–^). After 1 week, the P-treatments started with half of the tubes supplied with a low P nutrient solution (LP, 0.1 mmol. L^–1^ of KH_2_PO_4_^–^) and the other half with a high P nutrient solution (HP, 1 mmol. L^–1^ of KH_2_PO_4_^–^).

In LP, KNO_3_, NH_4_NO_3_, and KH_2_PO_4_ concentrations of 10.9 mmol. L^–1^, 0.1 mmol. L^–1^ and 0.1 mmol. L^–1^ were applied, respectively, and in the HP treatment, KNO_3_, NH_4_NO_3_ and KH_2_PO_4_ concentrations of 10 mmol. L^–1^, 1 mmol. L^–1^, and 1 mmol. L^–1^ were applied, respectively. The slight increase in the NH_4_^+^ concentration under HP should not have affected plant development and only had a marginal impact on solution Ec; NO_3_^–^-N was not limiting at a concentration of 11 mmol.L^–1^ in both treatments (Lecompte et al., 2013), and the NO_3_^–^/NH_4_^+^ ratio of 110 and 11 in the LP and HP treatments, respectively, was well above the levels at which the plant might show sensitivity to NH_4_^+^ as a source of N. Other macronutrients were kept constant in the two nutrients solutions, i.e., 3.5 Mg^2+^; 3.25 Ca^2+^ and 2.5 SO_4_^2–^ (in mmol. L^–1^), and micronutrients were also kept constant, i.e., 0.5 B, 0.02 Cu^2+^, 8.2 Fe^2+^, 0.5 Mn^2+^, 0.01 MoO_4_^2–^ and 0.1 Zn^2+^(in μmol. L^–1^).

Plants were grown for 3 weeks with these different nutrient solutions. All the plants, regardless of the genotype and P treatment, received the same amount of water required to maintain substrate water content during growth. Thus, total P supply was the product of P concentration in the nutrient solutions by the amount of irrigation water, and at harvest, the plants had received 43 mg and 4.3 mg of P in the HP and LP treatments, respectively.

### Root Extraction

At the end of the third week after the onset of P treatments, the taproot had almost reached the bottom of the tube and the plants were harvested. Each plant was carefully removed, first by submerging the tube in water-filled containers and then by gently tapping the tube, which was held at a 45° angle. The roots were separated from the substrate by washing above a sieve to prevent fine root loss. The nature of the substrate made it possible to separate the root system without causing damage. The aerial part of the plants was separated from the root systems and then weighed. Root and shoot sub-samples were dried in an oven at 75°C during 72 h to calculate their dry mass.

### Root Sampling and Scanning

**FIGURE 1:**
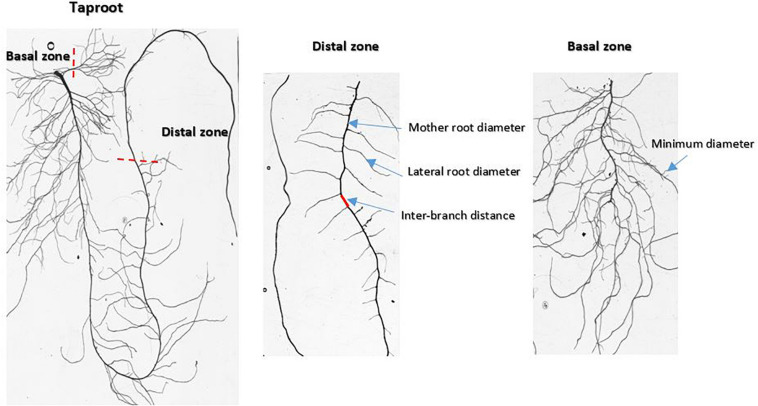
Images of scanned root systems illustrating the sampling zones and the positions of measurements carried out inside the root architecture.

Lettuce and wild-associated species have a root system organized around a taproot that grows downward. At harvest, older lateral roots may reach similar diameters and lengths to those of the taproot. Two regions of the root system were sampled to assess the different root traits (Figure 1): the lower part of the taproot in the distal zone and the basal zone, which provides information on fine roots. Fine roots are lateral roots of the highest branching order (usually 4th). Individual root samples of each plant were collected (total: 168 root samples) and scanned at 3,200 dpi (EPSON V750) in transparent mode. Scanned images were analyzed later with ImageJ software^1^ to measure the root architectural traits.

### Root Trait Measurements

At harvest, taproot depth (depth) was measured directly on the root using a ruler. This trait represents the maximum length reached by the taproot. The other root traits were measured on the images of the scanned samples using the measuring tools provided by the ImageJ software. Interbranch distance (ibd) was calculated as the average of forty-five measurements along the taproot in the distal zone starting from the first distal lateral root (Figure 1). The minimal diameter (dmin) was calculated as the average of thirty measurements performed on higher order lateral roots in the basal zone. The diameter is known to vary along the root axis (Wu et al., 2016). The maximal apical diameter (dmax) was computed as the 90th quantile in a distribution of diameters along the taproot in the distal zone. One value per plant was calculated.

The slope of the linear regression of lateral root diameters by their mother root (taproot) diameter (DlDm) was calculated for each plant based on forty-five measurements of diameters made of the mother root in the distal zone and its daughter roots, which are the first-order lateral roots. The line was forced to pass through the point of coordinates (dmin, dmin). A low DlDm reveals a high dominance of the taproot on the first-order laterals. Elongation activity (elong) provides information on the actual depth reached by a root of a given diameter during the experiment, and it is calculated for each plant as the ratio of depth by dmax and by the number of days since transplantation. Traits were calculated for each plant in the two treatments. Means for each genotype in the two P treatments were used to compute a relative response for assessing the phenotypical plasticity of the different traits:

$$\mathrm{Rtrait}\left(\%\right)=100^{*}[\left(\mathrm{trait}\mathrm{value}\mathrm{under}\mathrm{LP}\right)-$$

(traitvalueunderHP)/(traitvalueunderHP)].

### P Content and Efficiency

Dried plant tissues were ground into fine powder to measure the P concentration in the shoots and roots with a portable X-ray fluorescence spectrometer (XRF, S1 TITAN, Bruker Germany). This analyzer determines elemental concentrations by measuring the secondary fluorescent X-ray emitted by the collection upon excitation (Reidinger et al., 2012). XRF analysis calibrations were performed by comparison using the inductively coupled plasma-mass spectrometry (ICLPMS) method as a reference (Jenner et al., 1990). This calibration was carried out during a preliminary test and conducted on 25 collections in total, i.e., 15 leaf samples and 10 root collections. Although the correlation between the XRF and ICP measurements was satisfactory (*p* < 0.001, R^2^ = 0.93), the slope differed from 1. Therefore, a correction of the XRF dosage was uniformly applied to the whole data set presented here according to the linear adjustment.

PUE and its components were defined as:

PUpE=Pabsorbed(mg)/Papplied(mg).

PUtE=totaldrymass(mg)/Pabsorbed(mg).

PUE was calculated as PUpE x PUtE (mg total dry mass/mg P applied).

### Statistical Analysis

All statistical analyses were performed using R software^2^. Two-way analyses of variance (ANOVA) were performed to analyze the effects of the genotype (G), P level and their interactions (G x P) on the root traits. Means for each genotype were compared with Tukey’s test. The coefficient of variation (CV) is provided to describe the genetic variability for each trait in the LP and HP treatments. To provide additional information about the impacts of the treatments for each genotype, values in the HP and LP treatments were compared with a Wilcoxon-Mann-Whitney non-parametric test at a significance threshold of *p* = 0.01 because of the limited number of biological replicates.

To summarize the information regarding the response to low P and evaluate the association between traits, a principal component analysis (PCA) was performed, with the relative traits as variables. Correlations among the different variables were obtained by the r Pearson correlation coefficient. A second group of PCAs was performed to link root traits to PUE and its components under HP and LP.

## Results

### Genetic Variation of the Response of Traits to P Availability

**FIGURE 2:**
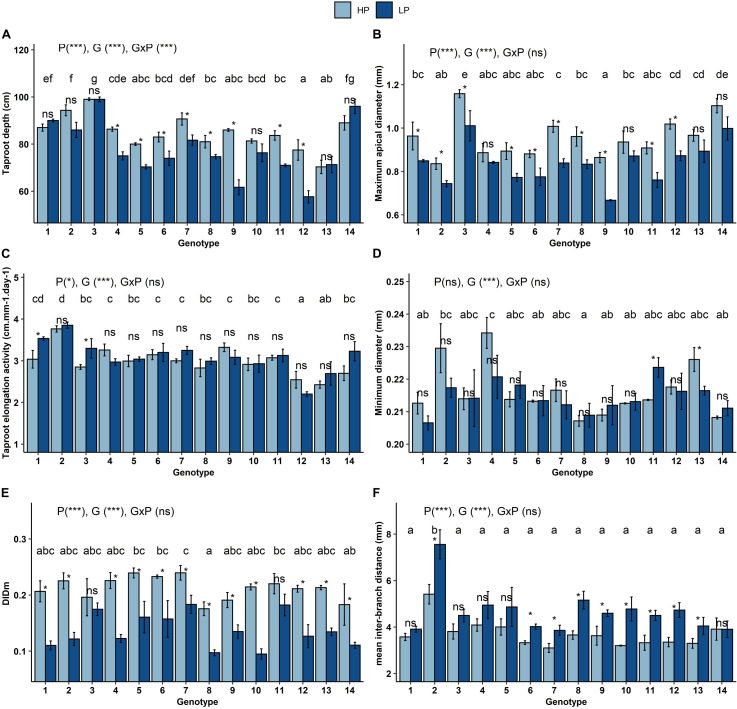
Mean (± standard error) value of the root architecture traits for 14 *Lactuca* genotypes grown at sufficient (HP) and limiting (LP) P availability: taproot depth **(A)**, maximum apical diameter **(B)**, taproot elongation activity **(C)**, minimum diameter **(D)**, linear regression slope of lateral root diameters by mother root diameter (DlDm) **(E)**, and inter-branch distance **(F)**. Each bar is the mean of three replications (corresponding to three plant per genotype). The significance of the *p*-value for each factor P, genotype, and genotype x P interaction is indicated for each trait (*p < 0.05; ***p < 0.001; ns: not significant). Different letters are used to indicate means that differ significantly (Tukey’s HSD, *p* = 0.05). Asterisk (*) indicates a significant difference between P conditions in the same cultivar using a Wilcoxon test (*p < 0.1; ns: not significant).

Two-way ANOVAs was carried out for each trait, and revealed an effect of genotype and P treatment on all the traits except dmin, which was not affected by P stress. Moreover, the genotype x P treatment interaction were not significant except for depth (Figure 2A). The results of Wilcoxon tests for the P effect on each individual genotype are given in Figure 2. Taproot depth at the time of excavation varied between 72 and 98 cm under HP (CV = 9.04%) and 58 and 100 cm under LP (CV = 16%). Depth was reduced by 8% on average under LP compared to HP; however, a strong genotype x P interaction was observed. Genotypes G9 (old butterhead cultivar), G11 (modern crisphead cultivar) and G12 (old crisphead cultivar) showed the strongest reduction in depth under LP (−28%, −15%, −25% respectively), while for the other genotypes, the root depth was more moderately reduced (G4, G5, G6, G7,G8) or did not change significantly (G1, G2, G3, G10, G13, G14) (Figure 2A).

Dmax ranged between 0.83 and 1.15 mm under HP (CV 10.77%) and between 0.66 and 1.01 mm under LP (CV 12.28%) (Figure 2B). Stem lettuce (G14) and wild *L. sativa* (G3) produced the thickest taproots, while genotypes G2 (*L. saligna*) and G9 (old butterhead cultivar) had the thinnest taproots. Dmax was reduced by 13% on average under LP compared to HP, although the Wilcoxon test for P treatment was not significant for the four genotypes.

For elong, regardless of the P treatment, the wild genotypes G1 and G2 showed the highest elongation potential and genotypes G12 and G13 showed the lowest elongation potential (Figure 2C). Elong increased slightly under LP by 5% compared to HP (*p* = 0.04), with the same range of variability among genotypes (CV of 14% under LP and 13% under HP).

Dmin was the trait that showed the least variation between genotypes (CV of 4.21% in HP and 3.69% in LP). Only two genotypes, G11 and G13, showed a significant increase or decrease in dmin under LP, respectively (Figure 2D).

DlDm was reduced under LP by 45% on average, indicating a stronger dominance of the taproot under LP (Figure 2E). The decrease in dmax was lower than that of DlDm, which also indicates that for most of the genotypes, the diameter of first-order lateral roots showed a strong decrease. Genotypic variation in DlDm was approximately twice as high under LP as under HP (27.44% vs 14.90%).

Ibd ranged from 3.85 to 7.55 mm under LP (CV 35%), and from 3 to 5.41 mm under HP (CV 23.47%) (Figure 2F). Genotype G2 (*L. serriola*) showed a higher ibd than the other genotypes. Ibd increased by 23% on average under LP compared to HP, although this increase was significant in only 9 genotypes out of the 14 studied.

### Relationship Between Root Architecture Traits in Response to Low P

**FIGURE 3:**
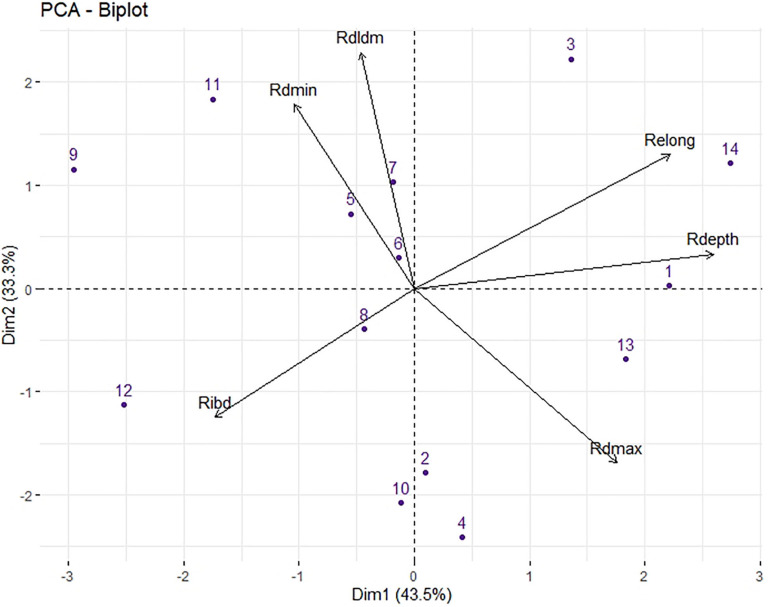
Principal component analysis (PCA) of six root traits for 14 *Lactuca* genotypes in response to low P: Rdepth, relative taproot depth; Rdmax, relative maximum apical diameter; Rdmin, relative minimum diameter; RDlDm, relative dominance between the mother and lateral root diameters; Relong, relative taproot elongation activity. Values are the means of the relative response calculated using the mean for each genotype in the two P treatments. The fourteen genotypes were identified by their numbers, as given in Table 1.

The two principal components (PCs) extracted by the PCA accounted for 76.8% of the variance (Figure 3), and the third component accounted for 11% and is not shown. Relative taproot depth (Rdepth) was strongly associated with component 1, while relative taproot dominance (Rdldm) and relative minimum diameter (Rdmin) were associated with component 2. Relative taproot elongation activity (Relong), Relative maximum diameter (dmax) and Relative inter-branch distance (Ribd) were associated with both components.

Correlations among relative responses of root traits to low P are given in Supplementary Table 1. Relong was positively correlated with Rdepth (*r* = 0.91, *p* < 0.001) (Supplementary Table 1), which is due to the calculation method of elong as the ratio of depth/(dmax^∗^time).

A positive correlation was also observed between Rdmax and Rdepth (*r* = 0.57, *p* < 0.01). Dmax and depth were correlated under LP (*r* = 0.52, Supplementary Figure 1) but not under HP (*r* = 0.19). Genotypes that showed the highest reduction in dmax at low P availability also showed strong reductions on their depth.

Depth and ibd were positively correlated in the HP group (*r* = 0.35, *p* < 0.05) but not in the LP group (*r* = −0.03). The PCA plot showed that Rdepth and Ribd were opposite in both component 1 and component 2 (Figure 3) and thus were negatively correlated (r = −0.52, *p* < 0.01, Supplementary Table 1).

This finding indicates that in response to low P, some genotypes maintained the depth of their main roots and did not greatly increase the distance between their lateral roots (G1, G3, G13, G14) while others (e.g., G10, G12) tended to decrease the elongation of their main roots while at the same time increasing the distance between lateral roots (Supplementary Figure 1A). We also observed a negative correlation between the relative DlDm (RDlDm) and Rdmax (*r* = −0.56, *p* < 0.05): the genotypes for which dmax responded weakly to low P, such as G4, G10, G13, and G14, showed a strong decrease in DlDm, and vice versa (Supplementary Figure 1B).

### Root Traits Associated With PUE

**FIGURE 4:**
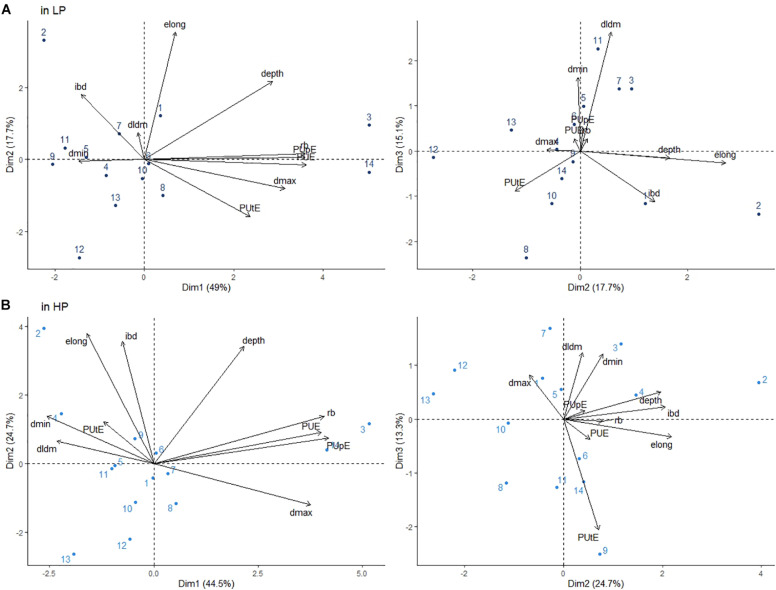
Principal component analysis (PCA) of root traits for 14 *Lactuca* genotypes located on the plane by components 1, 2, and 3 under LP **(A)** and HP **(B)**: rb: root biomass; depth, taproot depth; dmax, maximum apical diameter; dmin, minimum diameter; DlDm, dominance between the mother and lateral root diameters; elong, taproot elongation activity; and ibd: interbranch distance. Values shown represent the mean of three replicates in both the LP and HP treatments. See Table 1 for the genotype names.

The PUE value ranged from 16.27 gDM/gP to 69.92 gDM/gP under HP and 61.24 gDM/gP to 455.04 gDM/gP under LP (Supplementary Table 2). In both treatments, genotypes G3 (wild *L. sativa*) (455.03 gDM/gP and 68.83 gDM/gP under LP and HP, respectively) and G14 (stem lettuce) (454.26 gDM/gP and 69.92 gDM/gP under LP and HP, respectively) were the most efficient. To assess which traits were the most important determinants of PUE, a PCA was conducted on both treatments. Three components accounted for 82% of the variability in both cases (Figure 4). Similar to the LP treatment, PUE was very strongly associated with root dry mass along the first component in the HP treatment (Figure 4). This finding was confirmed by very high coefficients of correlation between the two variables: *r* = 0.93, *p* < 0.001 under HP and *r* = 0.98, *p* < 0.001 under LP. PUpE was also strongly associated with PUE, while PUtE was only correlated with PUE under LP. Two root traits, depth and dmax, were also associated with PUE, PUpE and root dry mass along the first component. In contrast, traits describing taproot dominance (DlDm), branching density (ibd) or thin root diameters (dmin) were not associated with PUE in either treatment.

**FIGURE 5:**
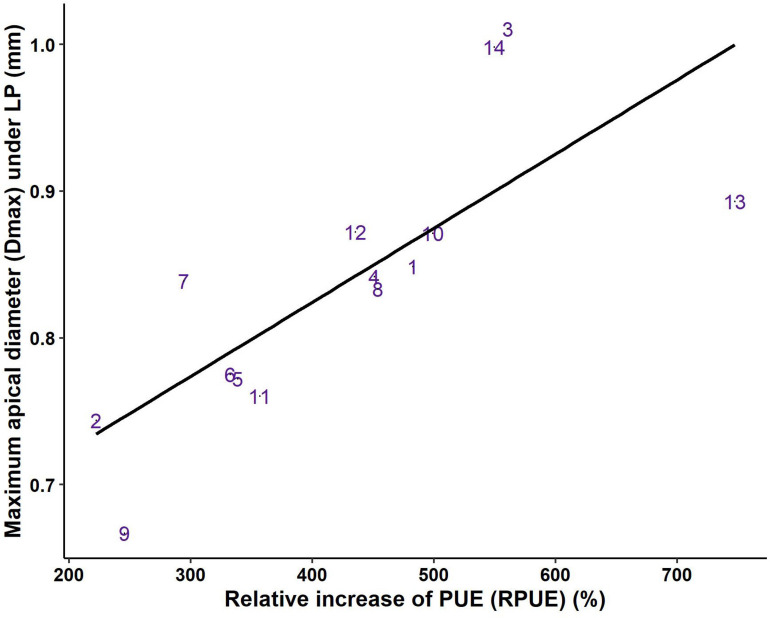
Relationship between the relative increase in P-use efficiency (RPUE) and maximum apical diameter (dmax) under low P availability (*r* = 0.76, *p* < 0.01). Values are the means of the relative response calculated using the mean for each genotype in the two P treatments. The fourteen genotypes are identified by their numbers, as given in Table 1.

The relative increase in PUE under LP compared to HP (RPUE) showed large variations between genotypes and ranged from 222% (G2, *L. saligna*) to 747% (G13, a modern Romaine lettuce), and RPUE was linearly associated with dmax under LP (Figure 5). Indeed, the highest values of RPUE were observed in genotypes with very large taproots (G3, G14), or in genotypes for which the decrease in dmax under LP was limited (G13). Accordingly, the lowest RPUE values were observed in genotypes with thin taproots and/or genotypes for which dmax was strongly reduced under LP (G2, G7, and G9) (Figure 5). This result emphasizes the importance of the maintenance of thick roots in *Lactuca* genotypes at low P availability to sustain root biomass and increase PUE.

## Discussion

The response of root system architectures to P deficiency may show differences beyond general tendencies and manifest variation between genera and species, or even within species. In this study, we investigated common patterns and specificities among a range of genotypes of *Lactuca* to identify the main architectural traits related to phosphorus-use-efficiency. Although the number of genotypes examined was limited to fourteen, this collection was sufficient to show substantial genetic variability for each trait and made it possible to estimate the range of responses to low P availability for each of these traits. The results emphasize the role of thick roots in the root system in such an adaptation and the increase in PUE at low P.

### Genetic Variation of the Root Architecture Traits in *Lactuca*

Six root traits were investigated: depth, dmax, elong, dmin, DlDm and ibd. These traits form useful descriptors of the root system architecture and its variability and they can summarize the developmental processes (e.g., elongation and branching) as well as the developmental characteristics (e.g., distribution of the root diameters) of roots, as previously shown in other studies that focus on intra- and interspecific root diversity (Pagès, 2014; Pagès and Picon-Cochard, 2014; Bui et al., 2015; Moreau et al., 2017; Salinier et al., 2019). These variables can also be used in modeling approaches such as the one proposed in the Archisimple model, which was elaborated by (Pagès et al., 2014).

Significant genetic variations were observed for all the different traits, and the magnitude of variations differed across the root traits. The genetic variability of depth and elong, which are correlated by construction, was not very important, although wild genotypes (G1, G2, and G3) to show a slightly greater depth and taproot elongation activity than the other *L. sativa* genotypes.

In a range of 45 species of dicots, Pagès (2014) observed a 4 to 5-fold variation of dmax ranging between 0.29 and 1.30 mm, and dmin ranging between 0.063 and 0.33 mm, depending on the species. According to Li et al. (2018), the average diameter in lettuce was approximately 0.5 mm. In our study, we characterized the variation in diameter by Dmax and Dmin. The values observed at high P availability, which ranged from 0.86 mm to 1.15 mm for dmax and 0.2 mm to 0.24 mm for dmin, fell within these limits. For dmax, the stem lettuce (G14) and the wild *L. sativa* (G3) produced thick taproots. For Dmin, low genetic variability was observed and the thinnest lateral roots occurred for either the wild, old or modern genotypes.

The values of ibd reported in this work are within the range of values observed for 60 species, including some belonging to the Asteraceae family (Pagès and Kervella, 2018). In another study on lettuce, the interbranch distance varied between 2.5 mm and 4 mm in *L. sativa* and *L. serriola* (Jackson, 1995), which corresponded to the values observed here. There was no significant difference among genotypes for ibd except for *L. saligna* (G2), which showed the highest ibd, i.e., the lowest branching density.

Describing taproot dominance defines the rate of diameter transition from the mother root to its laterals. A low value of DlDm denotes a high dominance of the taproot on first-order lateral roots. The values reported in this work correspond to those observed in the study of Pagès and Kervella (2018). The ancient romaine cultivar (G8) showed a low value of DlDm, i.e., a high dominance of the taproot.

Overall, a systematic pattern that could hierarchize the genotypes according to their varietal type or origin (ancient or modern) was not observed. However, wild genotypes sometimes showed some particularities, such as in depth, elong, dmax, and ibd.

### Effect of P Treatment on Root Architectural Traits

The analysis of the response of roots revealed a significant effect of P availability on all traits except dmin. General trends in response to P deficiency can be deducted from the absence of significant G × P interaction, except for depth.

P stress reduced the taproot depth, but the extent of reduction varied among genotypes (ranging from −11 to −55%). Butterhead cultivars (G4, G5, 9, and G12) showed the most pronounced reductions. In contrast, the root depths of wild species (G1, G2, and G3) were not significantly affected by low P. Whether or not this reveals a trend for wild species in natural soils to adapt to a large range of P availability requires further investigation. In a large collection of mungbean genotypes (153 genotypes), Reddy et al. (2020a) showed an increase in primary root length under low P. Sarker and Karmoker (2009) showed the same result in lentils and observed that increased root meristem volume was associated with an increase in root length under P-deficient conditions.

In this study, low P availability resulted in a reduction in dmax but did not affect dmin, indicating a reduction in the diameter range within the root system, and a shift toward lower diameters. Similarly, P deficiency decreased the average root diameter in beans (Beebe et al., 2006) and in oat (Zebrowska et al., 2017). Many papers have shown an increase in specific root length (SRL, the root length per unit mass) under P deficiency (Zhu and Lynch, 2004; Lynch and Brown, 2008), and it was associated with a decrease in root diameter (Lambers et al., 2006). However, contradictory observations were made on *Arabidopsis thaliana* L. (Heynh) where larger root diameters measured in the third apical segment were observed under low P (Ma et al., 2001).

Phenotypic plasticity was more pronounced for the traits relative to that of the branching pattern and dominance of the taproot, and ibd and DlDm were the most affected variables. DlDm was the most sensitive trait in response to P shortage and decreased by 45% in LP as compared to HP. Since dmax was reduced by only 13% on average, the lateral root diameters were more strongly affected than the taproot diameters. Lateral roots play an important role in P acquisition by increasing soil exploration and exploitation. As previously stated, the effects of P on the proliferation and growth of lateral roots seem to depend on the species (Borch et al., 1999; Mollier and Pellerin, 1999; Williamson et al., 2001; López-Bucio et al., 2002; Reymond et al., 2006). To our knowledge, no information has been previously published about the effect of P stress on lateral root density in lettuce. Here we measured an increase in ibd in lettuce under LP, i.e., a decrease in lateral root density. Similar results were observed in rice (Vejchasarn et al., 2016) and common bean (Borch et al., 1999).

In a previous study, we showed that the P deficiency imposed in this experimental set-up limited maximum photosynthesis and leaf area and thus the availability of carbon (C) for growth. Accordingly, the root biomass under LP was reduced in comparison to that under HP. The construction of fewer and thinner lateral roots, as observed in this study in response to low P, requires lower C. Since the growth and diameter of the taproot are less affected, it can be hypothesized that the reduction in C demand of the lateral root system allows the allocation of more of the remaining carbohydrates to the taproot. These results suggest that under P deficiency, lettuce genotypes maintain the extension of the taproot as much as possible to the detriment of the lateral root system. This adaptation was observed in a more or less pronounced way in all the genotypes studied. However, despite the absence of the genotype x P interaction, in the overall analysis of variance, we observed some genetic variability.

We observed a correlation between RDlDm and Rdmax (Supplementary Figure 1B), which indicates a trade-off between the maintenance of a thick taproot and taproot dominance in response to low P. Some genotypes maintained a thick taproot associated with increased taproot dominance, while for other genotypes, reductions in diameter were more homogeneous across root orders. Samadder et al. (2018) showed in soybean that the increase in the taproot length and taproot diameter leads to an expansion of the root absorptive surface and soil volume explored by the roots in soybean. As a result, plants can uptake more P.

A negative correlation was observed between the relative responses of ibd and depth (Figure 3 and Supplementary Table 1). This simultaneous evolution of depth and branching density at low P, which showed strong reductions for some genotypes and limited changes for others, is not a trade-off describing compensatory phenomena because some genotypes had structurally weakened root systems in terms of both elongation and branching. In particular, butterhead cultivars G9 and G12 either showed noticeably reduced root depth or increased ibd.

A range of other tradeoffs were observed among root functional traits across species (Ma et al., 2001; Walk et al., 2006; Wen et al., 2019; Honvault et al., 2020). According to Wen et al. (2019), root diameter is a good predictor of the root functional trait variation among species when P is limited. They showed that species with thinner roots relied on more branched lateral roots and higher specific root length to exploit more P in the soil. However, species with thicker roots were dependent on symbiotic mycorrhizal fungi for increasing P acquisition. In our study, the root diameter and lateral root density were investigated in the *Lactuca* genus under LP and HP; however, significant correlation were not observed between DlDm and ibd at either P level. This finding suggests that root system adaptations to low P vary depending on the species or families. Differences in root strategies are more important when several species are represented.

### Root Traits Contribute to the PUE

We observed that PUE was determined to a greater extent by PUpE than by PUtE, whether under LP or HP. PUE was highly correlated with root biomass under both LP and HP. The wild *L. sativa* genotype (G3 cv. PI251245) and the stem lettuce genotype (G14 cv. red orient) in particular showed a much higher root biomass and PUE compared to other genotypes. We explored the determinants of root biomass and their relationship with PUE at the two P levels. The two depth and dmax traits were most significantly correlated with root biomass and PUE, regardless of the P availability. PUE varied significantly among lettuce genotypes and showed important phenotypic plasticity in response to low P. The intensity of responses varied among genotypes, but we could not observe a pattern according to the origin or the type of the genotypes.

We identified that this relative variation in PUE under LP was primarily associated to dmax. Interestingly, a strong increase in PUE was observable in the genotypes that had the thickest taproots when P was not limiting as well as in the genotypes that showed the smallest decrease in dmax under low P, such as G10 and G13. Since the breeding effort should focus on genotypes that are efficient at low P availability, it might be interesting to study the genetic background that allows the maintenance of dmax under P stress.

These results are coherent with other works in which P-efficient genotypes exhibited large root systems in terms of root weight and length, which allowing these plants to explore a larger soil volume to acquire more P (Liu et al., 2004; Samadder et al., 2018). Additionally, Reddy et al. (2020b) showed that the root dry weight explained most of the variation of the PUE in mungbean and could be used as a pertinent indicator to breed P-efficient genotype.

However, PUE has been also related to other root architectural traits, such as lateral root density and lateral hair length and density. Since, the topsoil is often rich in P, the increase in root density in the upper part is an advantage for PUE (Zhu and Lynch, 2004; Hammond et al., 2009; van de Wiel et al., 2016). In addition, P-efficient genotypes in common bean were characterized by both denser and longer root hairs (Yan et al., 2004).

Because the root traits associated with increased PUE might depend on P distribution in the soil, the findings presented in this study should be extended to a wider context, such as to soils where horizontal extension of the roots would be less constrained while vertical root growth would be more restricted. However, the experimental setup presented in this study could reasonably be representative of a range of coarse-texture media and was suitable to explore the genetic diversity and identify traits associated with improved P efficiency.

## Conclusion

The present study showed that significant genotypic variations occurred in the root architectural traits in *Lactuca*, and they varied across root traits. DlDm and ibd had the highest genetic variation. Root trait plasticity was observed in response to low P availability, but there was no significant genotype x P interaction for most traits. Wild species had specific responses, particularly in terms of taproot depth and inter-branch distance. Overall, the lettuce genotypes showed increased primary root growth to the detriment of the branching process in response to low P. PUE was associated with root biomass under both P conditions. The maintenance of a large taproot diameter under low P appears to be the most pertinent trait for determining the relative increase in PUE in *Lactuca*. These results highlight the specific adaptations of the *Lactuca* genus under low P and provide information about the relevant traits to be conserved when selecting P-efficient varieties.

## Data Availability Statement

The raw data supporting the conclusions of this article will be made available by the authors, without undue reservation.

## Author Contributions

AB conducted experiment and data collection. AB, FL, and LP designed the experiment. AB, FL, AM, and LP analyzed the data. AB wrote the first draft of the manuscript. FL, AM, and LP revised the work in detail. All authors contributed to the manuscript revision and read and approved the submission version.

## Conflict of Interest

The authors declare that the research was conducted in the absence of any commercial or financial relationships that could be construed as a potential conflict of interest.

## Footnotes

1 http://rsbweb.nih.gov/ij/

2 https://www.R-project.org/

## Supplementary Material

Supplementary Figure 1 Correlations among the relative response to low P availability: **(A)** relative taproot depth (Rdepth) vs. relative inter-branch distance (Ribd) (*r* = −0.52, *p* < 0.01), **(B)** relative dominance between diameters of the taproot and its laterals (RDlDm) vs. relative apical diameter (Rdmax) (*r* = −0.56, *p* < 0.05). Values are the means of the relative response, calculated using the mean for each genotype in the two P treatments. The fourteen genotypes are identified by their numbers, as given in Table 1.
