# Supplementary figures and images for: Genetic Variation in Root Architectural Traits in Lactuca and Their Roles in Increasing Phosphorus-Use-Efficiency in Response to Low Phosphorus Availability
Source: Front Plant Sci. 2021 May 3;12:658321. doi: 10.3389/fpls.2021.658321 (PMC8128164; doi:10.3389/fpls.2021.658321)

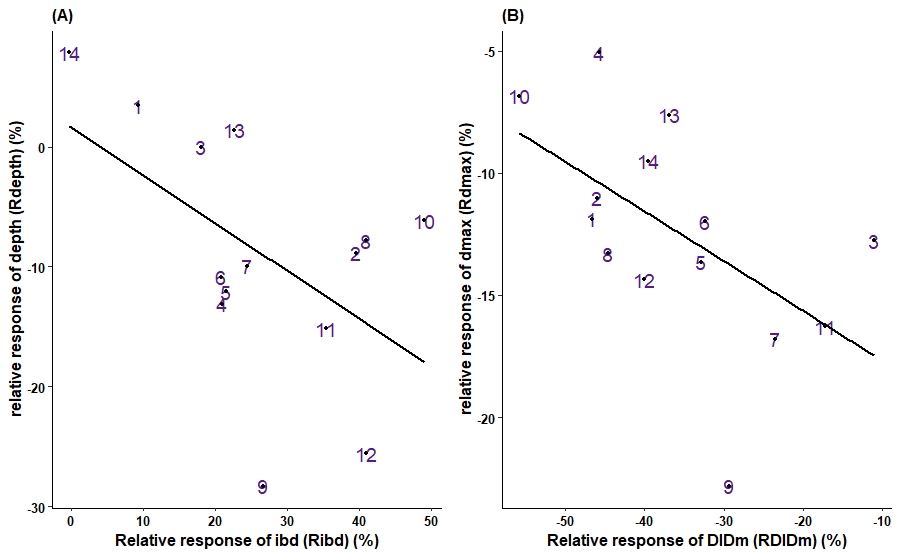

Supplement: Supplementary Figure 1 — Correlations among the relative response to low P availability: (A) relative taproot depth (Rdepth) vs. relative inter-branch distance (Ribd) (r = −0.52, p < 0.01), (B) relative dominance between diameters of the taproot and its laterals (RDlDm) vs. relative apical diameter (Rdmax) (r = −0.56, p < 0.05). Values are the means of the relative response, calculated using the mean for each genotype in the two P treatments. The fourteen genotypes are identified by their numbers, as given in Table 1. [file Image_1.JPEG]
