# Supplementary material for: Genetic Variation in Root Architectural Traits in Lactuca and Their Roles in Increasing Phosphorus-Use-Efficiency in Response to Low Phosphorus Availability
Source: Front Plant Sci. 2021 May 3;12:658321. doi: 10.3389/fpls.2021.658321 (PMC8128164; doi:10.3389/fpls.2021.658321)
Supplement: Supplementary file 2 [file Table_1.DOCX]

**Supplementary Table S1:** correlations among relative responses of root architecture traits.

Pearson correlation coefficients in bold type are significant at the 5% level (P <0.05).

|  | Rrb | Rdepth | Rdmax | Rdmin | RDlDm | Ribd | Relong |
| --- | --- | --- | --- | --- | --- | --- | --- |
| Rrb | 1 |  |  |  |  |  |  |
| Rdepth | **0.65*** | 1 |  |  |  |  |  |
| Rdmax | **0.58*** | **0.57*** | 1 |  |  |  |  |
| Rdmin | 0.001 | -0.20 | **-0.53*** | 1 |  |  |  |
| RDlDm | -0.11 | -0.08 | **-0.56*** | 0.44 | 1 |  |  |
| Ribd | -0.26 | **-0.53*** | -0.08 | 0.06 | -0.24 | 1 |  |
| Relong | **0.49*** | **0.91***** | 0.18 | 0.03 | 0.22 | **-0.59*** | 1 |

Rrb, relative root biomass; Rdepth, Relative taproot depth; Rdmax, relative apical maximum diameter; Rdmin, Relative minimum diameter; RDlDm, relative dominance between the diameters of mother and lateral roots; Ribd, relative inter-branch distance; Relong, Relative taproot elongation activity.
