# Supplementary material for: Genetic Variation in Root Architectural Traits in Lactuca and Their Roles in Increasing Phosphorus-Use-Efficiency in Response to Low Phosphorus Availability
Source: Front Plant Sci. 2021 May 3;12:658321. doi: 10.3389/fpls.2021.658321 (PMC8128164; doi:10.3389/fpls.2021.658321)
Supplement: Supplementary file 3 [file Table_2.DOCX]

**Supplementary Table S2:** PUpE, PUtE and PUE of 14 *Lactuca* genotypes grown under low P (LP) and sufficient P condition (HP).

| **P treatment** | **Genotypes** | **PUpE**  **Mean ± se** | | **PUtE**  **Mean ± se** | | **PUE**  **Mean ± se** | |
| --- | --- | --- | --- | --- | --- | --- | --- |
| **HP** | 13 | 0.085 | ± 0.013 | 192.64 | ±6.35 | 16,27 | ±1.89 |
|  | 2 | 0.1 | ± 0.017 | 200.6 | ±9.95 | 18,99 | ±2.56 |
|  | 12 | 0.13 | ± 0.01 | 170.33 | ±6.21 | 21,86 | ±1.86 |
|  | 1 | 0.15 | ± 0.026 | 159.19 | ±2.69 | 24,88 | ±4.58 |
|  | 4 | 0.12 | ± 0.016 | 214.98 | ±16.00 | 26,58 | ±3.35 |
|  | 11 | 0.15 | ± 0.057 | 231.25 | ±59.53 | 26,97 | ±7.90 |
|  | 10 | 0.16 | ± 0.018 | 184.82 | ±3.48 | 29,37 | ±3.95 |
|  | 5 | 0.18 | ± 0.009 | 181.01 | ±9.23 | 32,17 | ±1.04 |
|  | 7 | 0.20 | ± 0.026 | 164.64 | ±3.14 | 32,63 | ±3.89 |
|  | 8 | 0.18 | ± 0.008 | 189.01 | ±2.14 | 33,72 | ±1.97 |
|  | 9 | 0.15 | ± 0.036 | 236.97 | ±31.60 | 34,34 | ±3.43 |
|  | 6 | 0.23 | ± 0.005 | 212.23 | ±12.94 | 49,84 | ±1.75 |
|  | 3 | 0.43 | ± 0.054 | 159.16 | ±4.05 | 68,83 | ±9.45 |
|  | 14 | 0.33 | ± 0.016 | 213.15 | ±5.73 | 69,92 | ±4.76 |
| **LP** | 2 | 0.16 | ±0.046 | 401.29 | ±52.84 | 61,24 | ±10.85 |
|  | 12 | 0.25 | ±0.044 | 477.78 | ±44.07 | 117,05 | ±15.56 |
|  | 9 | 0.29 | ±0.056 | 414.32 | ±8.52 | 118,60 | ±20.84 |
|  | 11 | 0.30 | ±0.042 | 411.25 | ±10.13 | 123,25 | ±18.45 |
|  | 7 | 0.34 | ±0.040 | 387.23 | ±22.07 | 128,68 | ±10.25 |
|  | 13 | 0.32 | ±0.034 | 432.26 | ±8.77 | 137,98 | ±14.78 |
|  | 5 | 0.33 | ±0.047 | 428.02 | ±15.94 | 141,08 | ±18.86 |
|  | 1 | 0.37 | ±0.012 | 389.10 | ±27.77 | 144,96 | ±7.39 |
|  | 4 | 0.31 | ±0.022 | 470.27 | ±4.95 | 146,51 | ±10.48 |
|  | 10 | 0.40 | ±0.010 | 437.15 | ±10.54 | 175,96 | ±3.37 |
|  | 8 | 0.32 | ±0.043 | 574.32 | ±28.17 | 186,82 | ±33.07 |
|  | 6 | 0.45 | ±0.052 | 476.25 | ±15.95 | 215,50 | ±20.15 |
|  | 14 | 0.82 | ±0.055 | 553.76 | ±14.72 | 454,26 | ±33.00 |
|  | 3 | 0.89 | ±0.104 | 510.08 | ±13.84 | 455,03 | ±58.28 |

PUpE: Phosphorus uptake efficiency; PUtE: Phosphorus utilization efficiency; PUE: Phosphorus use efficiency.

Genotypes are ordered according to their PUE values.
